# Systems Biology ARDS Research with a Focus on Metabolomics

**DOI:** 10.3390/metabo10050207

**Published:** 2020-05-19

**Authors:** Sayed M. Metwaly, Brent W. Winston

**Affiliations:** 1 Department of Critical Care Medicine, Faculty of Medicine, University of Calgary, Calgary, AB T2N 4Z6, Canada; sayed.mohamedmetwaly@ucalgary.ca; 2 Departments of Medicine and Biochemistry and Molecular Biology, University of Calgary, Calgary, AB T2N 4Z6, Canada

**Keywords:** acute respiratory distress syndrome, biomarkers, metabolomics

## Abstract

Acute respiratory distress syndrome (ARDS) is a clinical syndrome that inflicts a considerably heavy toll in terms of morbidity and mortality. While there are multitudes of conditions that can lead to ARDS, the vast majority of ARDS cases are caused by a relatively small number of diseases, especially sepsis and pneumonia. Currently, there is no clinically agreed upon reliable diagnostic test for ARDS, and the detection or diagnosis of ARDS is based on a constellation of laboratory and radiological tests in the absence of evidence of left ventricular dysfunction, as specified by the Berlin definition of ARDS. Virtually all the ARDS biomarkers to date have been proven to be of very limited clinical utility. Given the heterogeneity of ARDS due to the wide variation in etiology, clinical and molecular manifestations, there is a current scientific consensus agreement that ARDS is not just a single entity but rather a spectrum of conditions that need further study for proper classification, the identification of reliable biomarkers and the adequate institution of therapeutic targets. This scoping review aims to elucidate ARDS omics research, focusing on metabolomics and how metabolomics can boost the study of ARDS biomarkers and help to facilitate the identification of ARDS subpopulations.

## 1. Introduction

**Figure 1:**
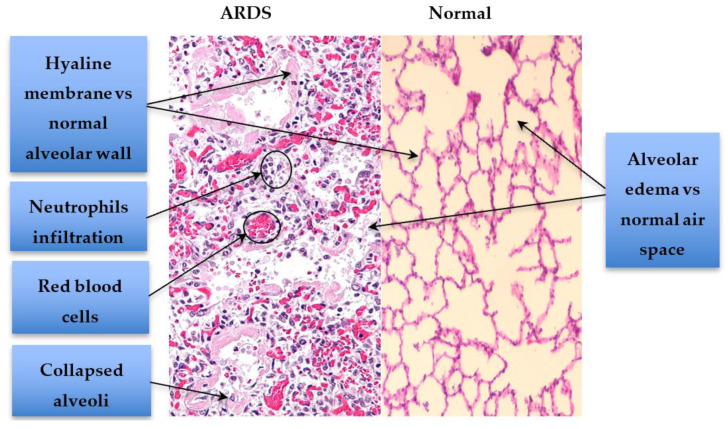
Exudative Diffuse Alveolar Damage (DAD) in early ARDS. Comparison between ARDS and normal lung tissue demonstrates the presence of alveolar collapse, neutrophil infiltration, areas of microscopic hemorrhage, hyaline membrane formation and alveolar edema in ARDS. Histopathology images were adapted and reproduced under Creative Commons licenses: ARDS histopathology component by Nephron (Own work) [CC BY-SA 3.0 (http://creativecommons.org/licenses/by-sa/3.0) or GFDL (http://www.gnu.org/copyleft/fdl.html)], via Wikimedia Commons; and Normal lung alveoli component By Jpogi —Own work, CC BY-SA 4.0, https://commons.wikimedia.org/w/index.php?curid=46568489.

Acute respiratory distress syndrome (ARDS) has consistently been an important cause of morbidity and mortality in the intensive care unit (ICU) [1]. Clinically, ARDS presents as acute hypoxic respiratory failure (i.e., arterial hypoxemia requiring oxygen supplementation, typically requiring an FiO_2_ of ≥ 0.50) developing within a week of exposure to a risk factor (commonly pneumonia or sepsis [2]), together with bilateral lung opacities on chest imaging [3]. In a recent study, age-adjusted ARDS incidence is estimated to be 27.6 cases per 100,000 person-years [4]. Patients surviving ARDS typically suffer long term sequelae, and the 3-year mortality rate following ARDS ranges from 44% to 71% [4]. There are still significant limitations surrounding the currently advocated Berlin definition of ARDS, namely the shortcomings of a clinical syndrome definition, limited knowledge about the basic etiology and mechanisms of the disease and a lack of a definitive clinico-pathologic correlate (e.g., only ~50% of biopsies from ARDS patients show diffuse alveolar damage (DAD) (Figure 1), a pathognomonic feature of ARDS [5,6]). Although the P_a_O_2_/F_i_O_2_ ratio is used to help identify ARDS, there is currently no biomarker to identify lung injury or to aid in making a diagnosis of ARDS. The utility of some clinically established biomarkers was explored in the setting of ARDS [7]. The use of B-type natriuretic peptide (BNP) was explored to help distinguish pulmonary edema secondary to congestive heart failure [8] from ARDS. Additionally, procalcitonin (PCT) was utilized as a differentiating marker between bacterial pneumonia and ARDS; however, it showed a relatively low sensitivity (~70%) [9]. This stresses the pressing need for discovering reliable ARDS diagnostic biomarkers (a true biomarker of lung injury). In this review, we will focus on omics research into ARDS susceptibility and diagnosis with a focus on metabolomics studies. We will also discuss aspects of ARDS heterogeneity and how omics research may help define ARDS heterogeneity.

## 2. Challenges in the Detection of ARDS and the Characterization of Its Susceptibility

Clinically, ARDS is very similar to an array of other conditions that cause arterial hypoxemia such as pneumonia, acute heart failure, diffuse alveolar hemorrhage, acute interstitial pneumonia and acute exacerbations of idiopathic pulmonary fibrosis [10]. While the Berlin definition sets forth objective criteria for diagnosing ARDS, importantly, the inter-observer discrepancy in interpreting the chest x-ray findings in keeping with ARDS remains concerningly high at 32% [11]. Additionally, direct measures of lung injury, by means of lung tissue histopathology, are too invasive to be of practical utility in ARDS diagnosis in the context of critically ill patients [10]. Of significant importance, as noted above, diffuse alveolar damage (DAD), the histopathological hallmark of ARDS, is detected in only about half of the tissue samples [5,6]. Thus, given the above, it is difficult to diagnose ARDS with certainty. In addition, typically, ARDS occurs following a risk factor, most commonly pneumonia and sepsis [4]. However, it is quite ironic that after five decades of relentless scientific research, we are still unable to predict why certain patients progress to ARDS following these common conditions and others do not. Taken together with the absence of a gold standard diagnostic test, it is readily recognizable why diagnosing ARDS early in the disease process can be fraught with challenges, especially in difficult cases.

## 3. Pre-clinical ARDS Research

ARDS animal models have been limited in their capacity to reproduce the full range of pathophysiological derangements occurring in ARDS [12]. Such limitations are largely related to differences in the physiology of animals used compared to humans [13]; for example, rat models have a heightened nitric oxide production response [14] and different innate immunity owing to their dissimilar Toll-like receptors [15], while sheep models have a higher susceptibility to lung injury following endotoxin challenge due to their pulmonary intravascular macrophages [16]. This explains the widespread poor outcomes encountered when translating interventions tested in animal ARDS models to humans [13]. Notwithstanding, very useful data about specific ARDS pathophysiological aspects can be gleaned when the results are carefully interpreted in the context of the distinct physiology of the animal model used and the nature of the intervention applied [13]. In particular, ARDS animal studies have elucidated the importance of two pathways that have attracted intense scientific interest, namely, inflammasomes and soluble receptor for advanced glycation end-products (sRAGE) [13]. Research has established the role of inflammasomes, intracellular protein complexes activated in cellular stress or infection, in triggering the innate immune response via the release of proinflammatory cytokines such as interleukin (IL)-1beta and IL-18 [17]. Using real-time polymerase chain reaction (RT-PCR), Dolinay and colleagues carried out comprehensive gene expression profiling on the whole blood of ARDS patients and found elevated levels of inflammasome-related mRNA transcripts (CASP1, IL1B and IL18) together with high levels of IL-18 [18]. The group further sought to experimentally prove the role of inflammasomes in developing ARDS using a mouse model. Both wild-type mice and mice genetically deficient in IL-18 or caspase-1 were mechanically ventilated (tidal volume 12 ml/kg) to induce ventilator-induced lung injury (VILI), a form of ARDS [18]. IL-18 levels increased upon mechanical ventilation, and interestingly, mice treated with IL-18-neutralizing antibody or genetically knocked out for IL-18 or caspase-1 exhibited reduced lung injury in response to mechanical ventilation [18]. Several independent studies confirmed the protective effect of IL-18 blocking pharmacotherapy or the gene deletion of caspase-1, IL-1β or IL-18 in protecting mice from hypoxia following exposure to high tidal volume mechanical ventilation [19], lipopolysaccharide (LPS) injection [19,20] or *Pseudomonas aeruginosa* injection [21].

Receptor for advanced glycation end-products (RAGE) is a pattern recognition receptor abundantly expressed on the membrane of type 1 alveolar epithelial cells [13]. Soluble receptor for advanced glycation end-products (sRAGE), on the other hand, shows consistent association with poor outcomes in ARDS and has been suggested as a marker of ARDS severity [13]. Jabaudon and colleagues found an inverse correlation between the level of sRAGE and pulmonary fluid clearance in an acute lung injury (ALI) model in mice [22]. In a follow-up study, the group experimentally established the effect of blocking membrane-bound RAGE by monoclonal antibodies, or injecting sRAGE in restoring alveolar fluid clearance and preventing hypoxia in an acid aspiration ALI model in mice [23]. 

Despite the limitations of ARDS animal models, careful set-up, thoughtful interpretation and adequate follow-up studies can elucidate and have elucidated important pathophysiological aspects of ARDS. 

## 4. ARDS Genomics and Transcriptomics Research

Genome-wide association studies (GWAS) looking into ARDS susceptibility have consistently failed to demonstrate significant associations between ARDS and specific loci [10,24]. Recently, a study by Reilly et al. suggested plasma angiopoietin-2 as a potential causal marker in indirect ARDS and its mortality. The study found five genetic variants in the ANGPT2 locus that are associated with higher levels of plasma angiopoietin-2 in people of European ancestry. However, in contradiction with Reilly and colleagues’ findings, several epidemiological studies previously confirmed evidence of a higher ARDS mortality rate in black and Hispanic rather than white patients [25,26]. Later on, in a more recent study, the group employed whole genome genotyping and Mendelian Randomization to establish the causal contribution of plasma sRAGE in sepsis-induced ARDS in people of European and African ancestry [27]. The study found a strong association between ARDS risk and plasma levels of sRAGE (odds ratio: 1.86, 95% confidence interval: 1.54–2.25) [27]. Due to the immense inflammatory reaction involved in ARDS, there has been continuous research interest in relating changes in cytokines to the detection and subclassification of ARDS [28,29,30,31,32,33,34], i.e., evidence of ARDS heterogeneity. Based on a series of studies, ARDS has been subclassified based on certain clinical features and the measured levels of cytokines into two subphenotypes, hypoinflammatory (uninflamed) and hyperinflammatory (reactive) [28,29,30,31,32,33,34]. In a pioneer study by Bos and colleagues, this approach was further enhanced by incorporating whole blood transcriptomics to look into the differences in mRNA expression between reactive and uninflamed subphenotypes of sepsis-induced ARDS [35]. Microarray technology identified differences in mRNA expression in 29% of the 11,334 genes studied. The reactive phenotype exhibited a higher expression of leucocyte activation genes, and pathway enrichment analysis highlighted the involvement of oxidative phosphorylation, mitochondrial dysfunction, cholesterol metabolism and the innate immune system. On the other hand, pathway analysis of the uninflamed phenotype showed the expression of the adaptive immune system and cell proliferation universally involved in cellular stress conditions. Though interesting, further study is required to ascertain the generalizability of these findings in ARDS precipitated by conditions other than sepsis. 

Importantly, these studies have shown that ARDS is not a uniform disease, that it is heterogeneous and that this heterogeneity may be explored to enrich for specific outcomes [36] and help predict responses to different therapies.

## 5. Metabolomics Studies of ARDS Detection

Metabolomics uses a systems biology approach to examine metabolites in an organism at a single point in time. This approach has demonstrated a remarkable capability for biomarker discovery. Generally, in metabolomics, small molecular weight compounds (<1000 daltons) are measured. These compounds include, but are not restricted to, sugars, amino acids, free fatty acids, small peptides, vitamins, steroids and xenobiotics [37]. Metabolites play essential roles in key cellular functions such as energy production and signal transduction. Hence, measuring metabolites can provide a valuable readout of cellular function and the interplay between hereditary makeup and the environment [38]. Metabolomics analytical methods have different compound specificities and lower detection limits (Table 1). Owing to the diverse classes of compounds measured and the wide variation in their concentration ranges in biological samples, applying more than one analytical method is increasingly common in research studies. A comprehensive coverage of the metabolomics analytical methods is beyond the scope of this review and can be found elsewhere [39,40,41]. The following text provides a brief overview of the metabolomics studies that have sought to identify ARDS diagnostic biomarkers.

In 1998, Schubert et al. used targeted GC-MS to compare nine metabolites in the exhaled breath of 19 ventilated ARDS patients and 18 ventilated surgical ICU (SICU) patients. ARDS patients showed significantly decreased levels of isoprene compared to ICU ventilated controls (median: 9.8 nmol/m^2^ per min, 95% confidence interval 8.2 ± 21.6 nmol/m^2^ per min in ARDS patients vs. median: 21.8 nmol/m^2^ per min, 95% confidence interval 13.9 ± 41.4 nmol/m^2^ per min in surgical ICU control patients, *p* = 0.04) [42]. Isoprene is a by-product of cholesterol synthesis [43], and it may exert a thermo-protective function in cells exposed to heat stress and may have a protective effect against reactive oxygen species [44]. However, the small sample size, lack of follow-up validation and difficulties associated with the reproducibility of GC-MS results make it hard to extrapolate the results to the extremely heterogeneous ARDS patient groups.

Similarly, in a study by the Bos group [45], untargeted GC-MS was employed to compare 500 metabolites in the exhaled breath of 42 ARDS patients and 52 ventilated ICU patients. Significant increases were seen in 3-methylheptane, octane and acetaldehyde levels in ARDS patients [45,46], a metabolomics pattern that reflects oxidative stress [47]. While Bos et al. used an appropriate control group and a larger sample size than Schubert group, there is a discrepancy in the metabolites associated with ARDS between both studies, which requires further follow-up validation. In addition, as a biomarker, the diagnostic accuracy is not particularly high (area under the curve (AUC), 0.80; 95% CI, 0.66–0.92) [45], and upon validation in a cohort of 19 ARDS patients and 27 controls, the identified biomarker exhibited a moderate diagnostic accuracy (AUC, 0.78; 95% CI, 0.65–0.91) [45]. Finally, the study could not differentiate mild from moderate/severe ARDS, i.e., it could not differentiate ARDS by severity.

In 2011, Stringer, et al. [48] used plasma ^1^H-NMR to assess the metabolic differences between 13 patients with sepsis-induced mild ARDS and six healthy controls. They identified significant increases in the levels of total glutathione, adenosine and phosphatidylserine and a decreased sphingomyelin level in ARDS patients compared to the healthy controls [48]. The identified metabolites reflect the involvement of a number of biological processes in ARDS pathogenesis, namely, oxidative stress (glutathione), energy metabolism (adenosine), apoptosis (phosphatidylserine) and the disruption of the endothelial barrier (sphingomyelin) [49,50,51]. A follow up study in 2014 used serum ^1^H-NMR to compare 14 ARDS patients and 33 unventilated sepsis patients and showed an association between ARDS and increased concentrations of phosphatidylserine, total lipids, total methylene lipids and total cholines [52]. This study was particularly novel as it identified the metabolite profile differences between ARDS and sepsis patients [52]. However, given the research evidence for the impact of mechanical ventilation on the metabolites [53,54], the use of control groups comprised of healthy non-ventilated individuals poses a potential important confounder in these studies. Additionally, the sample size was rather small.

In 2012, Rai and colleagues used ^1^H-NMR to compare metabolites in the non-bronchoscopic mini bronchoalveolar lavage fluid (mBALF) of 21 ARDS (10 moderate/severe ARDS and 11 mild ARDS) patients and nine ventilated ICU control patients [55]. The study showed significant differences in ethanol, alanine, betaine, proline, choline, threonine and lactate concentrations in controls versus ARDS (mild/moderate/severe) patients (R^2^Y = 0.89 and Q^2^ = 0.84). The study proposed the increased concentrations of branched chain amino acids (BCA), arginine, glycine, aspartic acid, succinate, glutamate and lactate and decreased concentrations of ethanol, acetate and proline as a metabolomics fingerprint associated with ARDS. Though interesting, the lack of validation limits the reliability of these findings. Additionally, the sample size was quite small, and mBALF may not be a practical sampling technique for large-scale metabolomics studies given the relative invasiveness of the procedure in ARDS patients. Finally, from a technical standpoint, bronchoalveolar lavage fluid is relatively scarce in metabolites apart from the lipid components of surfactant, and as such, LC-MS would have been a more appropriate platform to use in this study [56].

In 2014, the Evans group explored the metabolic differences in BALF samples of ARDS patients (*n* = 18) and healthy controls (*n* = 8) using LC-MS [56]. The study identified a higher level of lactic acid and purine base degradation metabolites (namely guanosine, xanthine and hypoxanthine), together with low phosphatidylcholine levels in ARDS patients [56]. This metabolomics fingerprint reflects the injury caused by inflammation [47] and oxidative stress [47]. However, Evans et al. used a healthy control group, the sample size was small, and there was no follow up validation.

In 2014, Singh et al. [57] performed a study using serum ^1^H-NMR to compare ARDS patients of various etiologies (*n* = 26) and mechanically-ventilated ICU controls (*n* = 19). The group indicated the higher occurrence of elevated *N*-acetylglycoproteins (NAC), acetoacetate, lactate, creatinine, histidine, formate and aromatic amino acids in ARDS patients. Singh et al. [57] used an appropriate control group, though the sample size was still relatively small.

In 2017, Rogers, et al. [58] used ultra-high-performance liquid chromatography/tandem mass spectrometry (UHLC/MS/MS) to compare the edema fluid of 16 ARDS patients and 13 controls with hydrostatic pulmonary edema [58]. The study could not differentiate between ARDS patients and the hydrostatic pulmonary edema controls [58].

In a series of two studies, Izquierdo-García and colleagues uniquely sought to differentiate ARDS secondary to influenza A pneumonia (*n* = 12) from patients with influenza A pneumonia (*n* = 18) [59], and ARDS secondary to *Streptococcus pneumoniae* pneumonia (*n* = 13) from *Streptococcus pneumoniae* pneumonia (*n* = 17) [60]. The group employed a 500 MHz NMR spectrometer for measuring serum metabolites in both studies. Interestingly, the group highlighted that ARDS patients show the same metabolic alterations despite the variation in the underlying etiology [60]. Though an interesting proof of concept, the sample sizes in both studies were not particularly high. 

In 2019, Lin and colleagues applied GC-MS to study the metabolic differences between plasma samples from 37 ARDS patients and 28 healthy controls [61]. The study identified significant differences in 128 out of 222 metabolites in ARDS patients. The group highlighted the affection of 92 pathways in ARDS patients when compared to the control group. Overall, the study had a relatively small sample size and a potential confounder due to the use of healthy rather than mechanically ventilated controls.

Although there have been several metabolomics studies of ARDS patients, it is difficult to make a definitive comment on the diagnostic metabopattern of ARDS patients because, in general, ARDS biomarker metabolomics studies to date have generally used unventilated controls, small sample sizes and/or have not validated the specific study findings [38,62].

## 6. Metabolomics Studies of ARDS Heterogeneity and/or Severity

Some of the above-mentioned ARDS detection metabolomics studies looked into disease heterogeneity and/or severity. Notably, in the above-mentioned study by Bos et al. [45], untargeted GC-MS was additionally employed to try to characterize ARDS severity and heterogeneity using volatile organic compounds (VOCs) in the exhaled breath of 42 ARDS patients. However, the group was not able to differentiate between direct and indirect ARDS (*p* = 0.24), or between mild, moderate and severe ARDS (*p* = 0.21) [45].

Stringer et al. [48] used plasma ^1^H-NMR for 13 patients with sepsis-induced ALI (mild ARDS) to study ARDS severity. However, the correlation between the acute physiology score (APS) and myoinositol (*r_s_* = 0.53, *q* = 0.25, *p* = 0.05), on one hand, and the correlation between APS and glutathione (*r_s_* = 0.56, *q* = 0.25, *p* = 0.04), on the other hand, were not strong [48].

Rogers et al. [58] hypothesized the presence of a subset of ARDS with a distinct metabolic profile [58]. They utilized UHLC/MS/MS to analyze the edema fluid of 16 ARDS patients. The group highlighted the presence of two ARDS metabolic subtypes, namely hypermetabolic and hypometabolic subtypes [30]. Contrarily to the hypometabolic ARDS (10/16, 62%) subtype, the hypermetabolic subtype was identified in six ARDS patients (6/16, 38%), who had higher concentrations of 235 metabolites and had ARDS mainly secondary to non-pulmonary sepsis. Pathway analysis indicated the significant impact of the alanine, aspartate and glutamate metabolic pathway [30]. Owing to the absence of plasma samples, the group was not able to compare their findings to Calfee et al.’s results [60].

In 2017, Viswan and colleagues utilized a high resolution 800-MHz ^1^H-NMR to characterize ARDS severity. Mini BALF (mBALF) samples (*n* = 36) were collected from patients with mild ARDS (*n* = 13) and moderate/severe ARDS (*n* = 23). The study successfully separated mild and moderate/severe ARDS with a 0.91 accuracy (R^2^Y = 0.72 and Q^2^ = 0.60) [63] using six metabolites (proline, lysine/arginine, taurine, threonine and glutamate). Pathway analysis highlighted the impaction of seven metabolic pathways, namely lysine biosynthesis; arginine and proline metabolism; taurine metabolism; aminoacyl-tRNA biosynthesis; glycine, serine and threonine metabolism; alanine, aspartate and glutamate metabolism; and D-glutamate metabolism [63]. However, an initial exploratory principal components analysis (PCA) scores scatterplot demonstrated a total overlap between mild and moderate/severe ARDS, contrary to the good separation noted for the partial least squares discriminant analysis (PLS-DA) model.

In the above-mentioned study by Lin and colleagues [61], plasma GC-MS identified significant differences in 128 out of 222 metabolites in ARDS patients (*n* = 37) and found a correlation between phenylalanine, aspartic acid and carbamic acid levels and ARDS severity grades [61]. They suggested a panel of four metabolites, namely ornithine, caprylic acid, azetidine and iminodiacetic acid, as a potential biomarker for ARDS severity [61]. Overall, the study had a relatively small sample size.

In 2019, Viswan and colleagues carried out another high resolution 800-MHz ^1^H-NMR study [64]. The study investigated ARDS *“subphenotype1”* (ARDS severity grades namely mild, moderate and severe ARDS) and *“subphenotype2”* (ARDS subtypes namely pulmonary and extrapulmonary ARDS) using both mBALF and serum samples [64]. The study could differentiate ARDS severity grades in the serum with good metrics (R^2^ = 0.80 and Q^2^ = 0.74) using 176 ARDS serum samples (mild *n* = 62, moderate *n* = 72 and severe *n* = 42). Additionally, the group distinguished ARDS severity grades in mBALF with good metrics (R^2^ = 0.77 and Q^2^ = 0.71) using 146 ARDS mBALF samples (mild *n* = 36, moderate *n* = 66 and severe *n* = 44). The study was also able to differentiate direct and indirect ARDS using ^1^H-NMR metabolomics with moderate metrics. In the serum (*n* = 147), the study was able to distinguish pulmonary (*n* = 67) and extrapulmonary (*n* = 80) ARDS based on metabolomics with R^2^ = 0.60 and Q^2^ = 0.48. Meanwhile, in mBALF (*n* = 128), the differentiation between pulmonary (*n* = 60) and extrapulmonary (*n* = 68) had R^2^ = 0.63 and Q^2^ = 0.51. The authors subdivided each of the previous groups into training and test sets, further looked into differentiating ARDS from controls, explored ARDS mortality and carried out pathway analysis. The overwhelming number of details provided, lack of explanation for the repeated variation in the number of samples used in each comparison and unclear study plan does not allow for conclusive findings; however, it does set the stage for the use of metabolomics in examining ARDS heterogeneity.

Unlike ARDS subphenotyping studies using cytokine assessment, there is no consensus agreement on the metabolic subclassification of ARDS. Most metabolomics studies have tried to explore the metabolic differences between clinically assigned pulmonary (direct) and extrapulmonary (indirect) ARDS with limited success. Importantly, no mechanistic explanation beyond a cursory pathway analysis has been provided for the essence of ARDS metabolic heterogeneity and the potential means for harnessing such knowledge for improving ARDS patients’ care in future translational research. The above-mentioned ARDS metabolomics studies are summarized in Table 2.

## 7. Future Directions

Defining the optimal sample type for studying ARDS has long been a matter of debate. On one hand, blood metabolites (e.g., those obtained from serum or plasma) represent the overall interaction between whole body biological systems and so are less specific than local lung metabolites (e.g., those obtained from the bronchoalveolar lavage fluid (BALF), lung edema fluid or exhaled breath condensate (EBC)) in studying ARDS. On the other hand, the local lung sampling techniques are more invasive, often less sensitive as they provide diluted samples that are less standardized, and less suitable for the repeated measures needed for prospective follow up [40]. We are yet to see an untargeted ARDS tissue metabolomics study that compares autopsy lung samples affected by diffuse alveolar damage (DAD) and lung samples of ICU controls. Such a study design is envisaged to provide the most specific metabolomics fingerprint of ARDS. Furthermore, if participants’ serum or plasma samples are concomitantly collected and analyzed by targeted metabolomics aiming to measure the levels of the identified tissue biomarker but in the serum or plasma, this would allow the timely identification of a highly specific biomarker that could be readily used for further prospective follow up validation studies. The pathway analysis of such a biomarker is likely to reflect specific biological processes involved in ARDS and, as such, help in understanding disease pathophysiology and may aid in developing specific therapies. 

Regarding ARDS metabolic heterogeneity, it is not surprising that metabolomics studies differentiating direct and indirect ARDS have universally reported low predictive ability. The sole reliance on a clinical predictor (ARDS etiology) as the standard for ARDS subclass assignment, together with the high misclassification rate of direct and indirect ARDS reported in the literature that can reach 37% [65], hugely undermines the predictive ability of any statistical model. The envisioned solution for this problem is to embrace a new, totally objective standard for ARDS subclassification and then explore the metabolic differences between subclasses or vice versa. A sensible approach would be to explore the metabolic differences between hypoinflammatory and hyperinflammatory ARDS subphenotypes, given their consistency and repeatability in several independent studies. Alternatively, a rather novel approach would be to devise a wholly new ARDS metabolic unsupervised subclassification and then examine how it correlates to ARDS clinical features such as responses to ventilator therapy and fluid therapy and overall survival. Either way, exploring the underlying biological processes that account for ARDS metabolic heterogeneity should be prioritized in aiming to unveil novel metabolic aspects of ARDS pathophysiology with the prospect of discovering potential therapeutic targets.

It is time to make a more coordinated effort to allow large studies to use metabolomics to aid in ARDS (lung injury) diagnosis and as an aid to determine ARDS subtypes. Even more importantly, these studies need to be validated using a diverse population of ARDS patients in a large validation study.

## Figures and Tables

**Table 1:** Strengths and weaknesses of nuclear magnetic resonance spectroscopy (NMR), gas chromatography mass spectrometry (GC-MS), and liquid chromatography mass spectrometry (LC-MS) analytical platforms ^1^.

Technique	NMR	GC-MS	LC-MS
Strengths	Non-destructive (several analyses can be conducted on the same sample) Minimal sample preparation Versatility for analyzing metabolites in biofluids, in tissues or in vivo High reproducibility and repeatability Quantifiable Extensive compound libraries	High spectral resolution Very sensitive/low limit of detection (ng) High mass accuracy for compound detection Reproducible retention times Highly developed compound libraries High separation efficiency	Ideal for thermostable and volatile and non-polar metabolites Can detect high molecular weight analytes Very high resolution; very sensitive (pg) lower detection limit Short separation time Simple sample preparation (derivatization not required) Detects a wider range of metabolites than GC-MS Very small sample size needed (~10 µL) Lipidomics variant allows for good detection of lipid metabolites
Weaknesses	Low sensitivity (only metabolites with relatively high concentrations (µg) can be detected) Overlap in peaks (different metabolites form peaks in the same regions)	Derivatization required (extensive sample preparation) Lower reproducibility (within and across labs) Fragmentation in MS (cannot re-use samples) Poor quantification Possible variation due to sample preparation	High solvent consumption and lower separation power Lower reproducibility (within and across labs) Ionization of metabolites (cannot re-use samples) Poor quantification for untargeted studies Poorly developed compound libraries compared to NMR and GC-MS

^1^ Adapted from [12,41].

**Table 2:** Metabolomics studies of acute respiratory distress syndrome (ARDS) detection, heterogeneity and severeity^1^. Surgical ICU, SICU; acute lung injury, ALI; mini bronchoalveolar lavage fluid, mBALF; branched chain amino acids, BCA; ultra-high-performance liquid chromatography, UHLC.

Authors	Study	Cases	Controls	Sample Type	Analytical Platform	Metabolites Profiled	ARDS Associated Metabolites
Schubert et al., 1998 [42]	ARDS detection	*n* = 19 ARDS	*n* = 18 ventilated SICU	Exhaled breath	GC-MS (targeted)	9	Isoprene
Stringer et al., 2011 [48]	ARDS detection and severity	*n* = 13 sepsis-induced ALI	*n* = 6 healthy	Plasma	1-H NMR	40	Total glutathione, adenosine, phosphatidylserine, sphingomyelin
Rai et al., 2012 [55]	ARDS detection	*n* = 21 ARDS	*n* = 9 ventilated ICU	mBALF	1-H NMR	>100	BCA, arginine, glycine, aspartic acid, succinate, glutamate, lactate, ethanol, acetate, proline
Evans et al., 2014 [56]	ARDS detection	*n* = 18 ARDS	*n* = 8 healthy	BALF	LC-MS	>500	Guanosine, xanthine, hypoxanthine, lactate, phosphatidylcholines
Bos et al., 2014 [45]	ARDS detection, heterogeneity and severity	*n* = 42 ARDS	*n* = 59 ventilated ICU	Exhaled breath	GC-MS (untargeted)	>500 (untargeted for test group) 5 for training and validation groups	3-methylheptane, octane, acetaldehyde
Singh et al., 2014 [57]	ARDS detection	*n* = 26 ARDS	*n* = 19 ventilated non-ARDS	Serum	1-H NMR	>100	*N*-acetylglycoproteins, acetoacetate, lactate, creatinine, histidine, formate, branched-chain amino acids
Stringer et al., 2014 [52]	ARDS detection	*n* = 14 ARDS	*n* = 33 unventilated sepsis	Serum	1-H NMR	51	Phosphatidylserine, total lipids, total methylene lipids, total cholines (in ARDS compared to sepsis)
Rogers et al. 2017 [58]	ARDS detection and heterogeneity	*n* = 16 ARDS	*n* = 13 hydrostatic pulmonary edema	Pulmonary edema fluid	UHLC/MS/MS2 for basic species, acidic species, and lipids.	760	235 were significantly higher in a subset of 6 ARDS patients (hypermetabolic)
Viswan et al., 2017 [63]	ARDS severity	*n* = 36 ARDS (23 moderate/severe ARDS and 13 mild ARDS)	None	mBALF	1-H NMR (high resolution, 800 MHz)	29	A proposed biomarker composed of six metabolites was identified. Proline, lysine/arginine, taurine and threonine were correlated to moderate/severe ARDS while glutamate was found to be characteristic of mild ARDS.
Izquierdo-García et al., [59]	ARDS detection	-Derivation set: *n* = 12 * -Validation set: *n* = 13 *	-Derivation set: *n* = 18 ** -Validation set: *n* = 13 **	Serum	1-H NMR (500 MHz)	N/A (spectral binning was applied, and only the significantly different bins were profiled)	ARDS patients have low serum glucose, alanine, glutamine, methylhistidine and fatty acid concentrations, and high phenylalanine and methylguanidine.
Izquierdo-García et al., [60]	ARDS detection	*n* = 13 ^#^	*n* = 17 ^##^	Serum	1-H NMR (500 MHz)	N/A (spectral binning was applied, and only the significantly different bins were profiled)	ARDS patients have low serum glucose, alanine, methylhistidine, fatty acids, citrate, creatine, creatinine and valine. Acetone levels are higher.
Viswan et al., 2019 [64]	ARDS heterogeneity and severity	Severity: - *n* = 176 serum - *n* = 146 mBALF Heterogeneity: - *n* = 147 serum - *n* = 128 mBALF	- *n* = 68 serum - *n* = 40 mBALF	Serum and mBALF	1-H NMR (high resolution, 800 MHz)	-54 in serum -52 in mBALF	-Serum: proline, glutamate, phenylalanine, valine -mBALF: isoleucine, leucine, valine, lysine/arginine, tyrosine, threonine
Lin et al., 2019 [61]	ARDS detection and heterogeneity	*n* = 37 ARDS	*n* = 28 healthy controls	Plasma	GC-MS	222	128 metabolites were significantly different in ARDS patients. There is a correlation between phenylalanine, aspartic acid and carbamic acid levels and ARDS severity grades. Ornithine, caprylic acid, azetidine and iminodiacetic acid, is a potential biomarker for ARDS severity.

^1^ adapted from [40]. * ARDS secondary to influenza A pneumonia, ** patients with influenza A pneumonia, ^#^ ARDS secondary to *Streptococcus pneumoniae* pneumonia; ^##^ patients with *Streptococcus pneumoniae* pneumonia.
